# Supplementary material for: CTM and QFD analysis: Framework for fintech adoption priority in commercial banks
Source: PLoS One. 2023 Nov 1;18(11):e0287826. doi: 10.1371/journal.pone.0287826 (PMC10619835; doi:10.1371/journal.pone.0287826)
Supplement: S1 Appendix — (DOCX) [file pone.0287826.s001.docx]

**Appendix. List of patents with the highest relation percentage for Topics 1 through 12**

**Table 1.** List of patents with the highest relation to Topic 1

| Title of Patent | Percentage | Publication Date | Applicant |
| --- | --- | --- | --- |
| User authentication | 85.50% | 2018-07-17 | BANK OF AMERICA CORPORATION |
| Customer identification verification process | 84.61% | 2019-06-11 | Capital One Services, LLC |
| Data transfer between self-service device and server over session or connection in response to capturing sensor data at self-service device | 82.59% | 2020-05-05 | BANK OF AMERICA CORPORATION |
| Enhancing information security using an information passport dashboard | 82.47% | 2017-08-29 | BANK OF AMERICA CORPORATION |
| Banking systems controlled by data bearing records | 80.80% | 2018-12-18 | BANK OF AMERICA CORPORATION |
| Parsing transaction information for extraction and/or organization of transaction-related information | 80.73% | 2019-11-26 | Capital One Services, LLC |
| Linking channel-specific systems with a user authentication hub to provide omni-channel user authentication | 80.47% | 2020-05-05 | Keys, Andrew T (Inventor) |
| Aggregation of automated teller machine (ATM) device-related information and/or factor-based selection of an ATM device | 80.38% | 2019-12-24 | Capital One Services, LLC |
| Processing of pre-staged transactions | 79.23% | 2016-08-30 | BANK OF AMERICA CORPORATION |
| Subjective route risk mapping and mitigation | 79.22% | 2018-07-17 | Allstate Insurance Company |

**Table 2.** List of patents with the highest relation to Topic 2

| Title of Patent | Percentage | Publication Date | Applicant |
| --- | --- | --- | --- |
| Method and system for processing a mobile payment transaction | 87.25% | 2018-08-14 | Apriva, LLC |
| System and method for authenticating, associating and storing secure information | 84.66% | 2020-01-07 | TENCENT TECHNOLOGY (SHENZHEN) COMPANY LIMITED |
| Point-of-sale and automated teller machine transactions using trusted mobile access device | 83.38% | 2014-11-11 | Schlesener, Matthew Carl (Inventor) |
| Securely storing and using sensitive information for making payments using a wallet application | 83.22% | 2020-10-06 | Minkasu, Inc |
| Controlling mobile payment transactions based on risk scores for point-of-sale terminals determined from locations reported by mobile terminals | 82.89% | 2019-06-18 | CA, Inc |
| Application system for mobile payment and method for providing and using mobile means for payment | 82.71% | 2019-12-10 | CELLUM GLOBAL |
| System and method for processing a beacon-based purchase transaction | 81.97% | 2016-03-29 | Apriva, LLC |
| Hands-free transactions verified by location | 81.73% | 2019-01-22 | Google Inc. |
| Enhanced security for digital wallets in multiple devices | 81.00% | 2020-08-18 | Wells Fargo Bank, NA. |
| Link of mobile devices to facilitate mobile commerce transactions | 80.14% | 2019-03-05 | International Business Machines Corporation (IBM) |

**Table 3.** List of patents with the highest relation to Topic 3

| Title of Patent | Percentage | Publication Date | Applicant |
| --- | --- | --- | --- |
| Automated digital method and system of providing or sharing access | 93.94% | 2020-09-15 | Middleware, Inc. |
| Efficient transfer of funds between accounts | 91.87% | 2018-07-17 | Green, Travis Harrison Kroll |
| Fraud prevention and replacement of credit/debit card lost, stolen, defective or fraudulently used | 90.26% | 2014-08-12 | Malek-Aslanian, Linda |
| Credit card supported electronic payment | 89.06% | 2011-08-23 | Kozee, Casey W. |
| System and method for providing transactional credit | 88.96% | 2014-07-01 | Bayne, Anthony Jeremiah |
| Methods and apparatus for facilitating a financial transaction | 88.61% | 2014-03-18 | Aton Behavioral Finance, LLC |
| Multi-purpose transaction account | 87.32% | 2012-01-24 | United Services Automobile Association (USAA) |
| Multi-purpose transaction account | 87.17% | 2014-03-04 | United Services Automobile Association (USAA) |
| Method and system for transferring electronic funds | 86.77% | 2011-10-04 | Ostrovsky, Vladimir |
| Systems and methods for mobile payments | 86.45% | 2014-12-30 | MShift Inc. |

**Table 4.** List of patents with the highest relation to Topic 4

| Title of Patent | Percentage | Publication Date | Applicant |
| --- | --- | --- | --- |
| Large liquidity seeking trading platform | 95.98% | 2020-05-12 | NYSE Group, Inc. |
| System and method for managing trading using alert messages for outlying trading orders | 95.94% | 2012-07-17 | CFPH, LLC |
| System and method for electronic trading | 95.93% | 2012-04-24 | Morgan Stanley |
| Method and system for internal matching | 95.44% | 2020-10-27 | Trading Technologies International, Inc. |
| Trading system with ELFs and umpires | 94.56% | 2013-02-19 | Stikine Technology, LLC |
| Methods and apparatus to internalize trade orders | 93.98% | 2020-02-18 | Trading Technologies International, Inc. |
| Automated trading system in an electronic trading exchange | 93.84% | 2014-05-13 | DCFB LLC |
| Accelerated trade matching using speculative parallel processing | 93.48% | 2014-10-21 | Chicago Mercantile Exchange Inc. |
| Trading system with price improvement | 93.42% | 2012-02-07 | BGC Partners, Inc |
| Controlling markets during a stop loss trigger | 93.33% | 2012-01-24 | Chicago Mercantile Exchange Inc. |

**Table 5.** List of patents with the highest relation to Topic 5

| Title of Patent | Percentage | Publication Date | Applicant |
| --- | --- | --- | --- |
| Automated banking system controlled responsive to data bearing records | 97.89% | 2016-06-21 | VanKeulen, Eric S. |
| Automated banking machine that operates responsive to data bearing records | 97.25% | 2012-03-13 | Diebold Self-Service Systems division of Diebold, Incorporated |
| Automated banking machine that operates responsive to data bearing records | 97.09% | 2012-02-28 | Diebold, Incorporated |
| Automated banking machine that operates responsive to data bearing records | 96.59% | 2011-08-02 | Diebold Self-Service Systems division of Diebold, Incorporated |
| Banking machine controlled responsive to data read from data bearing records | 96.26% | 2013-04-16 | Jenkins, Randall W. |
| Card activated cash dispensing automated banking machine authorization system and method | 96.17% | 2011-02-08 | Diebold Self-Service Systems division of Diebold, Incorporated |
| Banking system controlled responsive to data bearing records | 96.10% | 2014-04-01 | Diebold Self-Service Systems division of Diebold, Incorporated |
| Banking machine controlled responsive to data read from data bearing records | 96.06% | 2013-07-02 | Diebold Self-Service Systems division of Diebold, Incorporated |
| Automated teller machine with an encrypting card reader and an encrypting PIN pad | 96.05% | 2016-06-21 | Steinbach, Klaus |
| Banking system controlled responsive to data bearing records | 95.47% | 2014-05-27 | Diebold Self-Service Systems division of Diebold, Incorporated |

**Table 6.** List of patents with the highest relation to Topic 6

| Title of Patent | Percentage | Publication Date | Applicant |
| --- | --- | --- | --- |
| Method and system for converting an annuity fund to a life insurance policy | 95.21% | 2012-02-07 | Bankers Insurance Group, Inc. |
| Fund for hedging real estate ownership risk using financial portfolio theory and data feed for analyzing the financial performance of a portfolio that includes real estate | 92.81% | 2011-06-28 | Benda, Peter |
| Method and system for converting an annuity fund to a life insurance policy | 92.70% | 2012-02-07 | Bankers Insurance Group, Inc. |
| Method of operating a venture business | 89.83% | 2011-04-26 | Mintz, Harold P. |
| Methods and systems for providing interest rate indices and notes | 89.73% | 2014-01-28 | Barclays Capital Inc. |
| Investment fund for maximizing a risk adjusted expected return while providing a defined minimum income at maturity | 88.75% | 2014-02-04 | BlackRock Institutional Trust Company, National Association |
| Methods and systems for providing structured loan commitment transactions | 88.09% | 2011-09-06 | Barclays Capital Inc. |
| Machine, article, and method for an installment payment payout at contract value | 87.73% | 2014-03-25 | Concept Hedging, LLC |
| Annuity having interest rate coupled to a referenced interest rate | 87.13% | 2012-06-26 | Abbs, Donald Paul |
| Total fair value swap | 87.00% | 2011-07-26 | JP Morgan Chase Bank, NA |

**Table 7.** List of patents with the highest relation to Topic 7

| Title of Patent | Percentage | Publication Date | Applicant |
| --- | --- | --- | --- |
| Symmetric discovery over audio | 93.68% | 2020-09-01 | GOOGLE LLC |
| Method for the mutual authentication of entities having previously initiated an online transaction | 91.89% | 2019-12-10 | IDEMIA FRANCE |
| Method and arrangement for authorizing a user | 90.37% | 2019-09-24 | Mopper AB |
| Dynamic electronic communication with variable messages using encrypted quick response codes | 87.59% | 2020-11-17 | Capital One Services, LLC |
| Location augmented biotransaction systems and methods | 87.36% | 2018-06-19 | de Sylva, Robert Francis |
| Systems and methods for physical location verification | 87.03% | 2020-07-14 | Paypal, Inc. |
| Information providing apparatus and method, information processing apparatus and method, program storage medium, program, and information providing system | 86.29% | 2015-05-26 | Sony Corporation |
| Symbol-based communication routing | 85.91% | 2019-06-04 | State Farm Mutual Automobile Insurance Company |
| Fingerprint recognition control methods for payment and non-payment applications | 85.64% | 2017-05-02 | Egis Technology Inc. |
| Securities messages with automated encoding of field operators | 84.89% | 2019-07-23 | International Business Machines Corporation (IBM) |

**Table 8.** List of patents with the highest relation to Topic 8

| Title of Patent | Percentage | Publication Date | Applicant |
| --- | --- | --- | --- |
| Secure real-time processing of payment transactions | 86.66% | 2020-09-01 | Early Warning Services, LLC |
| Method and system for credits in a social network | 85.28% | 2020-03-24 | Mastercard International Incorporated |
| Payment real-time funds availability | 81.66% | 2020-11-17 | Weinflash, Laura |
| Payment real-time funds availability | 78.70% | 2020-09-08 | Early Warning Services, LLC |
| Method and system for linkage of blockchain-based assets to fiat currency accounts | 78.42% | 2018-07-17 | Mastercard International Incorporated |
| Systems for providing and processing surprise conditional gifts | 77.95% | 2019-09-10 | Capital One Services, LLC |
| Systems and methods for aggregating and managing financial service accounts | 77.88% | 2017-07-18 | Capital One Financial Corporation |
| Method and system for linked electronic wallet application | 76.68% | 2019-08-06 | Mastercard International Incorporated |
| System and method for determining social statements | 76.47% | 2019-09-17 | Capital One Services, LLC |
| Method and system for retry processing of controlled payment transactions | 75.64% | 2019-08-06 | Mastercard International Incorporated |

**Table 9.** List of patents with the highest relation to Topic 9

| Title of Patent | Percentage | Publication Date | Applicant |
| --- | --- | --- | --- |
| Managing insurance claim data | 92.34% | 2011-12-27 | Intuit Inc. |
| Systems and methods for providing coverage recommendation engine | 91.67% | 2014-01-21 | Hartford Fire Insurance Company |
| Insurance coverage and rating system and method | 90.34% | 2019-08-06 | State Farm Mutual Automobile Insurance Company |
| Identifying property usage type based upon smart sensor data | 89.70% | 2019-08-20 | Olander, Erin Ann |
| Managing insurance claim data across insurance policies | 88.49% | 2012-01-24 | Intuit Inc. |
| Deterministic pricing management of a portfolio of one or more applications | 88.29% | 2013-02-26 | Hewlett-Packard Development Company, LP. |
| System and method to determine an initial insurance policy benefit based on telematics data collected by a smartphone | 88.19% | 2015-05-19 | Hartford Fire Insurance Company |
| Product model filtered view | 85.02% | 2019-03-05 | Guidewire Software, Inc |
| Systems, computer-implemented methods, and computer medium to determine premiums and indemnities for supplemental crop insurance | 84.82% | 2014-04-01 | WATTS AND ASSOCIATES, INC |
| Insurance policy hold | 83.34% | 2013-10-22 | Guidewire Software, Inc. |

**Table 10.** List of patents with the highest relation to Topic 10

| Title of Patent | Percentage | Publication Date | Applicant |
| --- | --- | --- | --- |
| Method and system for a service provider to control exposure to non-payment by a service consumer | 94.66% | 2011-08-09 | Microsoft Corporation |
| Online car buying | 90.89% | 2012-06-19 | United Services Automobile Association (USAA) |
| Using configured application information to control use of invocable services | 89.50% | 2020-07-28 | Jain, Tushar |
| Integrated business system for web based telecommunications management | 88.91% | 2015-11-24 | James, Angela R. |
| Transaction coordinator for digital certificate validation and other services | 87.80% | 2014-08-26 | Hicks, Mack |
| Delivering customer specified receipt types at checkout | 85.62% | 2017-02-21 | Wal-Mart Stores, Inc |
| System for atypical third party channel utilization for resource distribution completion | 84.25% | 2019-10-01 | BANK OF AMERICA CORPORATION |
| System and method for providing customers with seamless entry to a remote server | 83.77% | 2013-05-07 | JPMorgan Chase Bank, NA. |
| Billing management package for internet access and web page utilization | 83.38% | 2013-08-13 | Rose, Edward M |
| Locating and organizing digital receipt data for use in in-store audits | 82.74% | 2014-01-21 | Wal-Mart Stores, Inc. |

**Table 11.** List of patents with the highest relation to Topic 11

| Title of Patent | Percentage | Publication Date | Applicant |
| --- | --- | --- | --- |
| Bicycle suspension control apparatus | 97.73% | 2013-04-23 | Shimano Inc. |
| Embedded touch POS machine | 97.12% | 2018-03-20 | Lin, Zhe |
| Cassette for automated teller machine | 96.03% | 2013-09-24 | Lee, Won Joon |
| Medium sensing apparatus, medium handling apparatus and financial device | 95.21% | 2015-09-08 | LG CNS Co., Ltd. |
| Method, apparatus, assembly and kit for identification token | 94.97% | 2012-05-01 | Mastercard International, Inc |
| Virtualization of a central processing unit measurement facility | 94.86% | 2016-09-20 | West, Jr., Patrick M. |
| Transaction terminal with signature capture offset correction | 94.85% | 2016-03-01 | Hand Held Products, Inc. |
| Single continuous belt in an escrow subassembly | 93.38% | 2020-05-26 | Diebold Nixdorf, Incorporated |
| Medium processing apparatus | 92.82% | 2014-09-30 | OKI ELECTRIC INDUSTRY CO., LTD. |
| Automated teller machine having a cassette apparatus | 92.82% | 2011-09-13 | Nautilus Hyosung Inc. |

**Table 12.** List of patents with the highest relation to Topic 12

| Title of Patent | Percentage | Publication Date | Applicant |
| --- | --- | --- | --- |
| Methods and systems for improving the underwriting process | 91.40% | 2019-11-26 | Massachusetts Mutual Life Insurance Company |
| Methods and systems for using multiple data sets to analyze performance metrics of targeted companies | 90.53% | 2013-02-19 | Jones, Justin A |
| Calculating predictive technical indicators | 89.84% | 2013-10-15 | PREDICTIVE TECHNOLOGIES GROUP, LLC |
| Consistent set of interfaces derived from a business object model | 89.19% | 2014-06-03 | SAP AG |
| Consistent set of interfaces derived from a business object model | 88.73% | 2014-12-30 | Adelmann, Stefan |
| Systems and methods for measuring relationships between investments and other variables | 88.26% | 2018-04-10 | Gerber, Sander |
| Consumer behaviors at lender level | 87.83% | 2011-01-25 | AMERICAN EXPRESS TRAVEL RELATED SERVICES COMPANY, INC. |
| Systems and methods for adjusting insurance workflow | 87.82% | 2014-11-18 | Hartford Fire Insurance Company |
| Methods and systems for retail customer referral compensation programs | 87.67% | 2013-10-29 | Motherlode Network |
| Finance function high performance capability assessment | 87.62% | 2012-06-19 | Accenture Global Services Limited |
