# Supplementary material for: CTM and QFD analysis: Framework for fintech adoption priority in commercial banks
Source: PLoS One. 2023 Nov 1;18(11):e0287826. doi: 10.1371/journal.pone.0287826 (PMC10619835; doi:10.1371/journal.pone.0287826)
Supplement: Attachment — Submitted filename: Response to Reviewer_20230401.docx [file pone.0287826.s002.docx]

**Original Manuscript ID:** PONE-D-22-32567

**Original Article Title:** “Patent-based contextual topic modeling and QFD analyses for commercial banks’ adoption priority for fintech”

**To:** PLOS One Access Editor

**Re:** Response to reviewers

Dear Editor,

Thank you for allowing a resubmission of our manuscript, with an opportunity to address the reviewers’ comments.

We are uploading (a) our point-by-point response to the comments (below) (*Response to reviewers*), (b) an updated manuscript with blue colors indicating changes (*Revised Manuscript with Track Changes*), and (c) a clean updated manuscript without blue color (*Manuscript).*

Best regards,

D. Kang and S. Sohn

**Reviewer#1, Concern #1:** Abstract: The abstract should sequence the objectives, techniques, and concise results. Revise accordingly.

**Author action:** As recommended, the abstract has been revised in the order of research objectives, methodologies, and results.

- As financial technology (fintech) is developing rapidly, many commercial banks experience difficulty deciding what kind of fintech to primarily focus on when managing their business. Owing to limited resources and assets, there is a practical need for guidelines for banks’ investments in fintech. This study provides a systemic procedure to identify promising fintech groups and their investment priorities. We propose a QFD-based decision support framework for banks by considering both aspects of the emerging fintech push identified using patent topic modeling and the market pull of banking services obtained from a survey of the literature and experts. An empirical application of the proposed QFD framework to major South Korean banks shows that transaction support technology, secure transactions, and trading platforms are the three most important fintech categories. The QFD results are utilized to guide individual banks for further investment strategies such as mergers and acquisitions, strategic partnerships, and spin-off operations. The proposed framework can be generalized and applied to other financial service firms.

**Reviewer#1, Concern #2:** Introduction: In the introduction section, clear research objectives are missing. This section should clarify the background, objectives, research gaps, and innovations. Be concise with the authors' research problem, gaps, objectives, and innovation contribution.

**Author action:** We thank the reviewer for constructive feedback. The introduction is revised to reflect the reviewer’s comments. Pleases refer to the Section 1 on pp. 3 – 5.

- **1. Introduction**

*… condensed*

This leads to potential ambiguity for many banking firms in setting up their plans to employ fintech to transform financial services to those that are cost-effective, convenient, reliable, and meet customer needs [7].

*… condensed*

Different technologies may be required to improve the services offered by banks. For example, to refine the loan interest rate, it is necessary to accurately estimate the default rate of borrowers. Deep learning and text-mining algorithms can be used for this purpose.

*… condensed*

Based on the aforementioned challenges, we provide a systemic procedure that can be used to identify promising fintech groups and their investment priorities. Our proposed Quality function deployment (QFD)-based decision support framework considers the fintech push and market pull of banking services. To consider the market pull aspect, we classified the services provided by commercial banks through the literature and expert surveys. For the fintech push, contextualized subject modeling is applied to patents to identify emerging fintech groups. We then applied the proposed framework to patent data filed at the United States Patent and Trademark Office (USPTO) with a survey of major South Korean commercial banks. Subsequently, fintech investment strategies are proposed based on the empirical results. This study uniquely contributes to banks’ efficiency in terms of fintech adoption by proposing an approach that fulfills research and practical needs.

*… condensed*

**Reviewer#1, Concern #3:** Literature review: Add more appropriate and advanced literature on commercial banking. You can get the idea from the following studies.

http://dx.doi.org/10.2139/ssrn.3107770; https://doi.org/10.1371/journal.pone.0270406

**Author action:** As recommended, the following references are included in the revised manuscript. Moreover, the literature review has been restructured and modified to help the readers understand research background to this study for the practical needs. The revision can be found in Section 2 on pp. 5 – 9.

- **2. Literature review**

This section reviews research on fintech adoption and the relationship between innovation and performance from the perspective of commercial banks.

**2.1. Choices of fintech adoption**

Fintech and its adoption in the banking industry have brought innovations in various financial services, such as credit services, deposit services, financial market trading and brokerage, financial product advisory and retail sales, and transfer and global remittance [14–18]. Based on the predicated positive influence of fintech adoption in the commercial banking business model, various suggestions have been made regarding the scope of choices. For example, Thakor [14] proposed the following four services for fintech innovation: (i) credit, deposit, and capital-raising services (i.e., crowdfunding, lending marketplaces, mobile banks, and credit scoring); (ii) payments, clearing, and settlement services (i.e., mobile wallets and digital exchange); (iii) investment management services (i.e., e-trading and robo-advice); and (iv) insurance services (i.e., data-driven risk pricing and contracts).

.*… condensed*

**2.2. Strategies of the fintech adoption**

The general adoption or investment choice for which financial services should be improved depends on technology readiness and service strategies. Therefore, commercial banks must consider appropriate investment strategies for a desirable outcome given the intended scope of the services. Recent trends in commercial banks’ efforts to acquire and develop intellectual capital can be explained from two perspectives: internal efforts (i.e., hiring data scientists and operating internal projects for innovation) and external efforts (i.e., funding or participating in joint ventures or mergers and acquisition of technology companies). Brandl and Hornuf [23] differentiated investments from three perspectives: full integration of another company, strategic partnership between firms, and spin-off operations led by banks with traditional business models. They identified that banks with traditional business models must consider the possibilities of technology-driven digitalized financial services and coordinate common technological standards and banking functions to realize appropriate performance.

*… condensed*

**2.3. Relationship between technological innovation and performance**

Technology-driven financial service development approaches produce innovative outcomes for new business models, applications, and process or products [29]. Consequently, commercial banks initiate and accelerate various types of research and development activities for patent acquisition. For example, Wang et al. [30] suggested that the levels of fintech innovation outcomes (reduction in bank operating costs, service efficiency improvement, strengthened risk control capabilities, and enhanced customer-oriented business models) depend on the bank’s use of technological innovation. Wang et al. [25] empirically validated that a positive impact of IT investments on intellectual capital can lead to competitive advantage, contingent on firm type, size, positioning, and location.

However, because of the nature of patents which take time to come into effect, commercial banks often need government support and selective investments for desirable outcomes. For example, investment-driven technological innovations require time to actualize and may not show immediate results at initial financing [31]. Investments in internal projects alone do not directly lead to investment performance, and government regulations must be considered for a fintech boom to become apparent [32]. Moreover, Haddad and Hornuf [33] noted that the availability of Internet server security, mobile subscription, and labor force also affect the development of fintech-driven markets. Based on various efforts to understand the relationship between the development of intellectual capital (such as patents), banking competitiveness, and performance, internal and external factors must be considered [25].

There remains a critical decision support question regarding the financial services that technology must be strategically applied to in an orderly fashion to appropriately fulfill target customer needs from commercial banks anticipating the digital transformation of traditional business models.

Please refer to Section 1 on p. 3 Line 55 - 57.

- Commercial banks, as a backbone of the banking system, are advised to integrate modern technologies to retain users and remain competitive [5,6].

**Reviewer#1, Concern #4:** Research methodology and data analysis. This study employs QFD methodology to derive “fintech priority” based on finance-related patent data and opinions collected from commercial bank practitioners. Methods are advanced and accurate. Add some detail that could justify the application of these methods in the study.

**Author action:** To fulfill the primary goal of the study (providing a decision-making support framework for banks considering dual perspectives of fintech push and market pull of banking services), the weights are incorporated by the ‘importance, urgency, and subjective.’ In addition to objective weights (in this case, ‘importance’ and ‘urgency’), Pang et al. (2020) emphasized the integration need for the subjective weights such as experts’ preferences and determination. While the current stage of the study examines the results based on these weights, the follow-up study examines the anticipated impact and its sensitivity relating to the weight information at different levels. To remain within the scope of the study, we therefore do not test out diversified approaches to the sub-measure of the weights.

- *Pang J, Guan X, Liang J, Wang B, Song P. Multi-attribute group decision-making method based on multi-granulation weights and three-way decisions. International Journal of Approximate Reasoning. 2020;117: 122–147. doi:*[*10.1016/j.ijar.2019.11.008*](https://doi.org/10.1016/j.ijar.2019.11.008)

**Reviewer#1, Concern #5:** Clarify the Sources of variable selection in table 1.

**Author action:** The table has been revised accordingly. Please refer to Section 3.1 on pp. 11– 12.

**Table 1.** Classification of financial services based on the literature

| Core financial services | Examples of services | Examples of technology disruption | Keywords | References |
| --- | --- | --- | --- | --- |
| Credit Services  (*i* = 1) | - Credit loan - Mortgage loan - Credit guarantee - Mobile banks, credit scoring | - Mobile credit loan - Crowdfunding - P2P lending | - Loan, - Lending - Credit - Guarantee | [17,23,37,38] |
| Deposit Services  (*i* = 2) | - Deposit or installment savings - Digital currencies - Mobile wallets | - Smart contracts in trade services and lending - P2P lending | - Deposit - Saving | [17,23,37,38] |
| Financial Market Trading & Brokerage  (*i* = 3) | - High-frequency trading - Copy trading - E-trading - Individual or group financial investments brokerage | - Social trading - Online brokerage | - Brokerage - Sales - Trading - Platform - Investment - Pricing | [17,23,38,39] |
| Financial Product Advisory & Retail Sales  (*i* = 4) | - Private banking services - Asset allocation advisory service - Trust services - Robo-advice | - Robo-advisory services - Branchless banking services | - Trust - Advisory - Advice | [17,23,38,40] |
| Transfer & Global Remittance  (*i* = 5) | - Letter of credit services - International trade settlements - Global remittances - Peer-to-peer transfer | - Global peer-to-peer money transfer of money in different countries - Blockchain-based markets - Foreign exchange applications | - Transfer - Remittance - Letter of credit - SWIFT | [23,38,40] |

**Reviewer#1, Concern #6:** Discussion. Add more references to interpret the results in sections 4.1, 4.2, and 4.3. Further, elaborate on the results in detail.

**Author action:** We thank the reviewer for valuable feedback. The results, implications for academic and practical needs are further elaborated through Table 8 in Section 4 on pp. 23 - 24.

- **4.2 Investment strategy for commercial banks**

*… condensed*

Finally, for Group III it is necessary to adopt conservative approaches in both investment and development strategies. Strategic partnership is recommended for the technology topics under this group, which will require a high level of collaboration for internal and external technology development for a desirable investment outcome. Results of the grouping and its implications are summarized in Table 8. We further interpret the abovementioned findings in the context of commercial banks in South Korea.

**Table 8.** Examples of degree of investment based on the partnership

|  | Full integration | Spin-off operations | Strategic partnership |
| --- | --- | --- | --- |
| Classification  (Examples) | Group I  (Topics 1, 3) | Group II  (Topics 2, 4, 5, 6, 11, 12) | Group III  (Topics 7, 8, 9, 10) |
| Technology details | Transaction security, Transaction support technology | Mobile transactions, trading platform, uncontacted financial service, Financial product valuation and design, hardware configuration, data analysis | Information and data processing, Personalized financial services, Insurance-related technology, Software for online services |
| Fintech service priority level (*y*-axis) | Moderate to High | Low to Moderate | Low |
| Fintech development status (*x*-axis) | Moderate to High | Moderate to High | Low |
| Development strategy characteristics | Time-sensitive involvements are required in both technology development and service integration as an immediate integration approach | The readiness of technology is considered relatively low. Instead of immediate application, the feasibility and assessments are recommended under collaborative efforts | Collaboration for both internal and external technology developments are recommended to for a synergistic performance |
| Degree of collaboration | High | Moderate | Low |
| Degree of investments | High | Moderate | Low |
| Examples in the South Korean context | Leading commercial bank in South Korea established Global Loyalty Network (“GLN”) which is a globally integrated platform that offers cross-border use of digital assets | Leading commercial bank in South Korea established a joint venture (JV) with medium size Tech company to lead the small and medium-sized business (SME) market by connecting financial ICT and technological competitiveness | Most large commercial banks in South Korea are incubating several technology startups for future growth |

**Reviewer#1, Concern #7:** Conclusion and future studies. Add limitations and future research ideas in the conclusion section. Give more policy implications for commercial banks.

**Author action:** The conclusion has been restructured and modified. The revision can be found in Section 5 on pp. 24 - 27.

- *… condensed*

Globally, particularly in South Korea, policymakers tend to separate the management of financial and nonfinancial corporations. Specifically, in the case of commercial banks, various direct or indirect regulations tend to disturb the rapid introduction of fintech by commercial banks. Most commercial banks worldwide do not speed up digital transformation because of regulations. For the long-term development of the financial system, it is necessary to quickly introduce developing fintech technology into traditional commercial banks, and active support from policymakers is required.

**Reviewer#1, Concern #8:** Avoid grammatical and typo errors and revise the manuscripts for these concerns.

**Author action:** We thank the reviewer for a careful attention to the details. The manuscript has been thoroughly checked for grammar errors by professionals from the Cactus Communication.

**Reviewer#1, Concern #9:** Improve the quality of Figure 1.

**Author action:** The figure has been revised as recommended. Please refer to Section 3 on p. 11.

**Fig 1.** The proposed framework

**Reviewer#2, Concern #1:** Abstract: This section is well organized; however, it must avoid grammatical and typo mistakes.

**Author action:** We thank the reviewer for a careful attention to the details. Some of the contents of the abstract have been changed and thoroughly revised. The manuscript has been thoroughly checked for grammar errors by professionals from the Cactus Communication.

- As financial technology (fintech) is developing rapidly, many commercial banks experience difficulty deciding what kind of fintech to primarily focus on when managing their business. Owing to limited resources and assets, there is a practical need for guidelines for banks’ investments in fintech. This study provides a systemic procedure to identify promising fintech groups and their investment priorities. We propose a QFD-based decision support framework for banks by considering both aspects of the emerging fintech push identified using patent topic modeling and the market pull of banking services obtained from a survey of the literature and experts. An empirical application of the proposed QFD framework to major South Korean banks shows that transaction support technology, secure transactions, and trading platforms are the three most important fintech categories. The QFD results are utilized to guide individual banks for further investment strategies such as mergers and acquisitions, strategic partnerships, and spin-off operations. The proposed framework can be generalized and applied to other financial service firms.

**Reviewer#2, Concern #2:** Introduction: The problem statement and innovations need to be clearer and more detailed.

**Author action:** We thank the reviewer for constructive feedback. The newly revised introduction is revised to meet the following standards: clarification of background, research objectives (problem statement), gaps in academic and managerial approaches, and contributions (innovations). Pleases refer to the Section 1 on pp. 3 – 5.

- **1. Introduction**

*… condensed*

This leads to potential ambiguity for many banking firms in setting up their plans to employ fintech to transform financial services to those that are cost-effective, convenient, reliable, and meet customer needs [7].

*… condensed*

Different technologies may be required to improve the services offered by banks. For example, to refine the loan interest rate, it is necessary to accurately estimate the default rate of borrowers. Deep learning and text-mining algorithms can be used for this purpose.

*… condensed*

Based on the aforementioned challenges, we provide a systemic procedure that can be used to identify promising fintech groups and their investment priorities. Our proposed Quality function deployment (QFD)-based decision support framework considers the fintech push and market pull of banking services. To consider the market pull aspect, we classified the services provided by commercial banks through the literature and expert surveys. For the fintech push, contextualized subject modeling is applied to patents to identify emerging fintech groups. We then applied the proposed framework to patent data filed at the United States Patent and Trademark Office (USPTO) with a survey of major South Korean commercial banks. Subsequently, fintech investment strategies are proposed based on the empirical results. This study uniquely contributes to banks’ efficiency in terms of fintech adoption by proposing an approach that fulfills research and practical needs.

*… condensed*

**Reviewer#2, Concern #3:** Literature review: Technological factor impact on commercial banks should be discussed more in the literature section. Add more advanced literature on the topic.

**Author action:** The literature review has been restructured and modified to help the readers understand research background to this study focusing on the role of technological factor impact on commercial banks. The revision can be found in Section 2 on pp. 5 – 9.

- **2. Literature review**

This section reviews research on fintech adoption and the relationship between innovation and performance from the perspective of commercial banks.

**2.1. Choices of fintech adoption**

Fintech and its adoption in the banking industry have brought innovations in various financial services, such as credit services, deposit services, financial market trading and brokerage, financial product advisory and retail sales, and transfer and global remittance [14–18]. Based on the predicated positive influence of fintech adoption in the commercial banking business model, various suggestions have been made regarding the scope of choices. For example, Thakor [14] proposed the following four services for fintech innovation: (i) credit, deposit, and capital-raising services (i.e., crowdfunding, lending marketplaces, mobile banks, and credit scoring); (ii) payments, clearing, and settlement services (i.e., mobile wallets and digital exchange); (iii) investment management services (i.e., e-trading and robo-advice); and (iv) insurance services (i.e., data-driven risk pricing and contracts).

.*… condensed*

**2.2. Strategies of the fintech adoption**

The general adoption or investment choice for which financial services should be improved depends on technology readiness and service strategies. Therefore, commercial banks must consider appropriate investment strategies for a desirable outcome given the intended scope of the services. Recent trends in commercial banks’ efforts to acquire and develop intellectual capital can be explained from two perspectives: internal efforts (i.e., hiring data scientists and operating internal projects for innovation) and external efforts (i.e., funding or participating in joint ventures or mergers and acquisition of technology companies). Brandl and Hornuf [23] differentiated investments from three perspectives: full integration of another company, strategic partnership between firms, and spin-off operations led by banks with traditional business models. They identified that banks with traditional business models must consider the possibilities of technology-driven digitalized financial services and coordinate common technological standards and banking functions to realize appropriate performance.

*… condensed*

**2.3. Relationship between technological innovation and performance**

Technology-driven financial service development approaches produce innovative outcomes for new business models, applications, and process or products [29]. Consequently, commercial banks initiate and accelerate various types of research and development activities for patent acquisition. For example, Wang et al. [30] suggested that the levels of fintech innovation outcomes (reduction in bank operating costs, service efficiency improvement, strengthened risk control capabilities, and enhanced customer-oriented business models) depend on the bank’s use of technological innovation. Wang et al. [25] empirically validated that a positive impact of IT investments on intellectual capital can lead to competitive advantage, contingent on firm type, size, positioning, and location.

However, because of the nature of patents which take time to come into effect, commercial banks often need government support and selective investments for desirable outcomes. For example, investment-driven technological innovations require time to actualize and may not show immediate results at initial financing [31]. Investments in internal projects alone do not directly lead to investment performance, and government regulations must be considered for a fintech boom to become apparent [32]. Moreover, Haddad and Hornuf [33] noted that the availability of Internet server security, mobile subscription, and labor force also affect the development of fintech-driven markets. Based on various efforts to understand the relationship between the development of intellectual capital (such as patents), banking competitiveness, and performance, internal and external factors must be considered [25].

There remains a critical decision support question regarding the financial services that technology must be strategically applied to in an orderly fashion to appropriately fulfill target customer needs from commercial banks anticipating the digital transformation of traditional business models.

**Reviewer#2, Concern #4:** Research methodology and data analysis. This section is properly organized. But still contain grammatical and typo mistakes. Revise it

**Author action:** We thank the reviewer for a careful attention to the details. The manuscript has been thoroughly checked for grammar errors by professionals from the Cactus Communication.

**Reviewer#2, Concern #5:** Discussion. More citations are required to back your study results in the discussion section. Find appropriate literature to strengthen your results.

**Author action:** We thank the reviewer for valuable feedback. The results, implications for academic and practical needs are further elaborated through Table 8 in Section 4 on pp. 23 - 24.

- **4.2 Investment strategy for commercial banks**

*… condensed*

Finally, for Group III it is necessary to adopt conservative approaches in both investment and development strategies. Strategic partnership is recommended for the technology topics under this group, which will require a high level of collaboration for internal and external technology development for a desirable investment outcome. Results of the grouping and its implications are summarized in Table 8. We further interpret the abovementioned findings in the context of commercial banks in South Korea.

**Table 8.** Examples of degree of investment based on the partnership

|  | Full integration | Spin-off operations | Strategic partnership |
| --- | --- | --- | --- |
| Classification  (Examples) | Group I  (Topics 1, 3) | Group II  (Topics 2, 4, 5, 6, 11, 12) | Group III  (Topics 7, 8, 9, 10) |
| Technology details | Transaction security, Transaction support technology | Mobile transactions, trading platform, uncontacted financial service, Financial product valuation and design, hardware configuration, data analysis | Information and data processing, Personalized financial services, Insurance-related technology, Software for online services |
| Fintech service priority level (*y*-axis) | Moderate to High | Low to Moderate | Low |
| Fintech development status (*x*-axis) | Moderate to High | Moderate to High | Low |
| Development strategy characteristics | Time-sensitive involvements are required in both technology development and service integration as an immediate integration approach | The readiness of technology is considered relatively low. Instead of immediate application, the feasibility and assessments are recommended under collaborative efforts | Collaboration for both internal and external technology developments are recommended to for a synergistic performance |
| Degree of collaboration | High | Moderate | Low |
| Degree of investments | High | Moderate | Low |
| Examples in the South Korean context | Leading commercial bank in South Korea established Global Loyalty Network (“GLN”) which is a globally integrated platform that offers cross-border use of digital assets | Leading commercial bank in South Korea established a joint venture (JV) with medium size Tech company to lead the small and medium-sized business (SME) market by connecting financial ICT and technological competitiveness | Most large commercial banks in South Korea are incubating several technology startups for future growth |

**Reviewer#2, Concern #6:** Conclusion and future studies. Revise the conclusion to describe your study's problem, methods, and innovative findings. Further research topics related to this framework could be discussed here.

**Author action:** The conclusion has been restructured and modified. The revision can be found in Section 5 on pp. 24 - 27.

**Reviewer#3, Concern #1:** The practical significance of the paper is poor. Whether financial practitioners, regulators or scholars, it is generally agreed that financial security is the most important issue in the financial industry, and the contribution of FinTech to this issue is mainly reflected in transaction technology and information processing capabilities. The authors seem to have just clarified a common sense through complicated means and a lot of work, which results in the lack of valuable policy implications in the paper.

**Author action:** Whilst various articles emphasize “financial security” as a vital issue in the financial industry, there remains a practical issue with related to the application process from strategic investment perspective. Specifically, while recent waves of digital innovations have led to a positive outlook for various fintech application projects, traditional and commercial bank managers and executives are faced with challenging tasks in deciding how to prioritize investments and developments (Jafarzadeh et al. 2022). To that end, this study aims to deliver an integrative approach to identifying key financial services and their orders based on both academic and practitioner’s perspectives. The practical significance and implications are further elaborated on Table 8 on p. 23.

- *Jafarzadeh H, Heidary-Dahooie J, Akbari P, Qorbani A. A project prioritization approach considering uncertainty, reliability, criteria prioritization, and robustness. Decision Support Systems. 2022;156: 113731. doi:*[*10.1016/j.dss.2022.113731*](https://doi.org/10.1016/j.dss.2022.113731)
- **4.2 Investment strategy for commercial banks**

*… condensed*

Finally, for Group III it is necessary to adopt conservative approaches in both investment and development strategies. Strategic partnership is recommended for the technology topics under this group, which will require a high level of collaboration for internal and external technology development for a desirable investment outcome. Results of the grouping and its implications are summarized in Table 8. We further interpret the abovementioned findings in the context of commercial banks in South Korea.

**Table 8.** Examples of degree of investment based on the partnership

|  | Full integration | Spin-off operations | Strategic partnership |
| --- | --- | --- | --- |
| Classification  (Examples) | Group I  (Topics 1, 3) | Group II  (Topics 2, 4, 5, 6, 11, 12) | Group III  (Topics 7, 8, 9, 10) |
| Technology details | Transaction security, Transaction support technology | Mobile transactions, trading platform, uncontacted financial service, Financial product valuation and design, hardware configuration, data analysis | Information and data processing, Personalized financial services, Insurance-related technology, Software for online services |
| Fintech service priority level (*y*-axis) | Moderate to High | Low to Moderate | Low |
| Fintech development status (*x*-axis) | Moderate to High | Moderate to High | Low |
| Development strategy characteristics | Time-sensitive involvements are required in both technology development and service integration as an immediate integration approach | The readiness of technology is considered relatively low. Instead of immediate application, the feasibility and assessments are recommended under collaborative efforts | Collaboration for both internal and external technology developments are recommended to for a synergistic performance |
| Degree of collaboration | High | Moderate | Low |
| Degree of investments | High | Moderate | Low |
| Examples in the South Korean context | Leading commercial bank in South Korea established Global Loyalty Network (“GLN”) which is a globally integrated platform that offers cross-border use of digital assets | Leading commercial bank in South Korea established a joint venture (JV) with medium size Tech company to lead the small and medium-sized business (SME) market by connecting financial ICT and technological competitiveness | Most large commercial banks in South Korea are incubating several technology startups for future growth |

**Reviewer#3, Concern #2:** Another fatal deficiency is why the US patent data should be used, rather than that of South Korea (if any). This leads to all your conclusions may be imprecise. The authors need to explain the relationship between American technology and South Korean FinTech applications or the relationship between American patent data and South Korean banking.

**Author action:** With respect to development and its patent registrations, USPTO has been gaining attentions for its lead in the global trend (Ailia et al. 2020). In the recent study of Liu and Qiao (2020), USPTO demonstrated a significant degree of leadership in the patent subject and proportion of profitable patents as well. Therefore, we selected USPTO as an appropriate representation of financial technology development trend. Also, the reason we use patents is to analyze world-leading technologies, not to reflect the needs of the Korean market, so we decided not to focus on patents registered with the Korean Intellectual Property Office. The revision can be found on Section 3.2. p. 12 Line 247 - 252.

- *Liu R, Qiao Y. Research on the patent subject profitability based on the big data analysis. J Phys: Conf Ser. 2020;1656: 012019. doi:*[*10.1088/1742-6596/1656/1/012019*](https://doi.org/10.1088/1742-6596/1656/1/012019)
- *Ailia MJ, Thakur N, Abdul-Ghafar J, Jung CK, Yim K, Chong Y. Current Trend of Artificial Intelligence Patents in Digital Pathology: A Systematic Evaluation of the Patent Landscape. Cancers. 2022;14: 2400. doi:*[*10.3390/cancers14102400*](https://doi.org/10.3390/cancers14102400)
- **3.2 Classification of fintech categories (HOW)**

*… condensed*

Concerning development and patent registrations, the USPTO has been gaining attention for its leadership in the global trend [38]. In a recent study by Liu and Qiao [39], the USPTO demonstrated a significant degree of leadership in the patent subject and proportion of profitable patents. Therefore, we collected patents filed at USPTO, an appropriate representation of the fintech development trend, with IPC codes G06Q 20 or G06Q 40 from 1972 to 2020, as displayed in Figure 2.

**Reviewer#3, Concern #3:** Are the research objects selected by the authors broadly representative? As far as the world's major economies are concerned, banking is a competitive industry, and banks of different sizes often have different business strategies. Therefore, the authors only select the four largest banks in South Korea, and the rationality of this need to be fully demonstrated.

**Author action:** Four case firms are selected as they are the only nationwide company which offers overseas branches with global recognition. We also specifically included financial groups with headquarter located in Korea as to secure accuracy of practitioners’ weight evaluations which are carried out in Section 3.1. The explanation is added in Section 3 on p. 10 Line 214 – 223.

- **3. Research methodology and data analysis**

*… condensed*

As case companies for commercial banks, four of the largest banks ranked by asset value in South Korea were utilized in this study. Namely, Woori, Shinhan, KB, and Hana financial groups are the only nationwide companies headquartered in South Korea. These firms have approximately over 305 billion USD (400 trillion KRW) total assets with over 25,000 employees. They represent traditional and commercial banks in South Korea for their historic establishment, going back to the early 1900s, with hundreds of branches in South Korea and global branches in other countries. These firms offer retail, corporate, and international banking; credit card operations; foreign exchange; and other services. Other commercial banks with different headquarters but available in South Korea are excluded from the study for the accuracy of the classification process by field practitioners (Section 3.1).

**Reviewer#3, Concern #4:** Please notes that the introduction section plays an important role in attracting attention. When the introduction is bad, the reviewer is likely to reject the paper. As a Research Article, the authors should particularly emphasize the contribution of this research to the body of knowledge. Unfortunately, I have not seen the relevant discussion.

**Author action:** We thank the reviewer for constructive feedback. The newly revised introduction is revised to meet the following standards: clarification of background, research objectives (problem statement), gaps in academic and managerial approaches, and contributions (innovations). Pleases refer to the Section 1 on pp.3 – 5.

- **1. Introduction**

*… condensed*

This leads to potential ambiguity for many banking firms in setting up their plans to employ fintech to transform financial services to those that are cost-effective, convenient, reliable, and meet customer needs [7].

*… condensed*

Different technologies may be required to improve the services offered by banks. For example, to refine the loan interest rate, it is necessary to accurately estimate the default rate of borrowers. Deep learning and text-mining algorithms can be used for this purpose.

*… condensed*

Based on the aforementioned challenges, we provide a systemic procedure that can be used to identify promising fintech groups and their investment priorities. Our proposed Quality function deployment (QFD)-based decision support framework considers the fintech push and market pull of banking services. To consider the market pull aspect, we classified the services provided by commercial banks through the literature and expert surveys. For the fintech push, contextualized subject modeling is applied to patents to identify emerging fintech groups. We then applied the proposed framework to patent data filed at the United States Patent and Trademark Office (USPTO) with a survey of major South Korean commercial banks. Subsequently, fintech investment strategies are proposed based on the empirical results. This study uniquely contributes to banks’ efficiency in terms of fintech adoption by proposing an approach that fulfills research and practical needs.

*… condensed*

**Reviewer#3, Concern #5:** In Section 4.2, the authors need to make a reasonable explanation for the grouping of topics. Why are they grouped in this way? What is the basis for dividing them into these three groups? Is it reasonable? Of course, the author did not explain why commercial banks should adopt different investment strategies for different groups.

**Author action:** Pleases refer to the Section 4.2 Table 8 on p.8.

**Table 8.** Examples of degree of investment based on the partnership

|  | Full integration | Spin-off operations | Strategic partnership |
| --- | --- | --- | --- |
| Classification  (Examples) | Group I  (Topics 1, 3) | Group II  (Topics 2, 4, 5, 6, 11, 12) | Group III  (Topics 7, 8, 9, 10) |
| Technology details | Transaction security, Transaction support technology | Mobile transactions, trading platform, uncontacted financial service, Financial product valuation and design, hardware configuration, data analysis | Information and data processing, Personalized financial services, Insurance-related technology, Software for online services |
| Fintech service priority level (*y*-axis) | Moderate to High | Low to Moderate | Low |
| Fintech development status (*x*-axis) | Moderate to High | Moderate to High | Low |
| Development strategy characteristics | Time-sensitive involvements are required in both technology development and service integration as an immediate integration approach | The readiness of technology is considered relatively low. Instead of immediate application, the feasibility and assessments are recommended under collaborative efforts | Collaboration for both internal and external technology developments are recommended to for a synergistic performance |
| Degree of collaboration | High | Moderate | Low |
| Degree of investments | High | Moderate | Low |
| Examples in the South Korean context | Leading commercial bank in South Korea established Global Loyalty Network (“GLN”) which is a globally integrated platform that offers cross-border use of digital assets | Leading commercial bank in South Korea established a joint venture (JV) with medium size Tech company to lead the small and medium-sized business (SME) market by connecting financial ICT and technological competitiveness | Most large commercial banks in South Korea are incubating several technology startups for future growth |

**Reviewer#3, Concern #6:** The abstract is unclear. The author needs to sort out the logic and expression of the abstract.

**Author action:** As recommended, the abstract has been revised in the order of research objectives, methodologies, and results.

- As financial technology (fintech) is developing rapidly, many commercial banks experience difficulty deciding what kind of fintech to primarily focus on when managing their business. Owing to limited resources and assets, there is a practical need for guidelines for banks’ investments in fintech. This study provides a systemic procedure to identify promising fintech groups and their investment priorities. We propose a QFD-based decision support framework for banks by considering both aspects of the emerging fintech push identified using patent topic modeling and the market pull of banking services obtained from a survey of the literature and experts. An empirical application of the proposed QFD framework to major South Korean banks shows that transaction support technology, secure transactions, and trading platforms are the three most important fintech categories. The QFD results are utilized to guide individual banks for further investment strategies such as mergers and acquisitions, strategic partnerships, and spin-off operations. The proposed framework can be generalized and applied to other financial service firms.

**Reviewer#3, Concern #7:** I did not see the relevant data in the Supporting Information files.

**Author action:** To analyze patents, we used the “patstat” database. Please understand that patent source data cannot be uploaded to Supporting Information files due to licensing issues. The explanation to the readers is clarified in Section 3.2. p. 15 Line 278 – 282.

- Subsequently, each topic was classified based on technology by comprehensively considering the relevant topic words and technologies included when reading the abstract of a patent (see Appendix A; The availability of detailed datasets can be discussed upon request under the consideration of license status. List of patents with the highest relation percentage for topics 1 through 12).

**Reviewer#3, Concern #8:** In line 201 of the PDF document, “These firms have approximately over 305 billion USD (400 trillion KRW) with more than 25,000 employees.”, where “305 billion USD” is a loan, deposit, or total asset?

**Author action:** The correction has been made in Section 3. p. 9 Line 216 – 218.

- These firms have approximately over 305 billion USD (400 trillion KRW) total assets with over 25,000 employees.

**Reviewer#3, Concern #9:** The authors lack the necessary explanation of the content to be proved by the figures and tables.

**Author action:** We thank the reviewer for a careful attention to the details. We add explanations for figures and tables.

**Reviewer#4, Concern #1:** The author needs to polish their manuscript in language.

**Author action:** We thank the reviewer for a careful attention to the details. The manuscript has been thoroughly checked for grammar errors by professionals from the Cactus Communication.

**Reviewer#4, Concern #2** The current innovations are not convincing. They are more likely to express the functions. It is suggested to rewrite the innovation point.

**Author action (1):** As recommended, we modified introduction part. Pleases refer to the Section 1 on pp. 3 – 5.

- **1. Introduction**

*… condensed*

This leads to potential ambiguity for many banking firms in setting up their plans to employ fintech to transform financial services to those that are cost-effective, convenient, reliable, and meet customer needs [7].

*… condensed*

Different technologies may be required to improve the services offered by banks. For example, to refine the loan interest rate, it is necessary to accurately estimate the default rate of borrowers. Deep learning and text-mining algorithms can be used for this purpose.

*… condensed*

Based on the aforementioned challenges, we provide a systemic procedure that can be used to identify promising fintech groups and their investment priorities. Our proposed Quality function deployment (QFD)-based decision support framework considers the fintech push and market pull of banking services. To consider the market pull aspect, we classified the services provided by commercial banks through the literature and expert surveys. For the fintech push, contextualized subject modeling is applied to patents to identify emerging fintech groups. We then applied the proposed framework to patent data filed at the United States Patent and Trademark Office (USPTO) with a survey of major South Korean commercial banks. Subsequently, fintech investment strategies are proposed based on the empirical results. This study uniquely contributes to banks’ efficiency in terms of fintech adoption by proposing an approach that fulfills research and practical needs.

*… condensed*

**Author action (2):** As recommended, the expected innovation outcomes driven by fintech are further elaborated. The literature review has been restructured and modified to help the readers understand the research background, such as driving factors of innovation performance of commercial banks, to this study for the practical needs. The revision can be found in Section 2 on pp. 5 – 9.

- **2. Literature review**

This section reviews research on fintech adoption and the relationship between innovation and performance from the perspective of commercial banks.

**2.1. Choices of fintech adoption**

Fintech and its adoption in the banking industry have brought innovations in various financial services, such as credit services, deposit services, financial market trading and brokerage, financial product advisory and retail sales, and transfer and global remittance [14–18]. Based on the predicated positive influence of fintech adoption in the commercial banking business model, various suggestions have been made regarding the scope of choices. For example, Thakor [14] proposed the following four services for fintech innovation: (i) credit, deposit, and capital-raising services (i.e., crowdfunding, lending marketplaces, mobile banks, and credit scoring); (ii) payments, clearing, and settlement services (i.e., mobile wallets and digital exchange); (iii) investment management services (i.e., e-trading and robo-advice); and (iv) insurance services (i.e., data-driven risk pricing and contracts).

.*… condensed*

**2.2. Strategies of the fintech adoption**

The general adoption or investment choice for which financial services should be improved depends on technology readiness and service strategies. Therefore, commercial banks must consider appropriate investment strategies for a desirable outcome given the intended scope of the services. Recent trends in commercial banks’ efforts to acquire and develop intellectual capital can be explained from two perspectives: internal efforts (i.e., hiring data scientists and operating internal projects for innovation) and external efforts (i.e., funding or participating in joint ventures or mergers and acquisition of technology companies). Brandl and Hornuf [23] differentiated investments from three perspectives: full integration of another company, strategic partnership between firms, and spin-off operations led by banks with traditional business models. They identified that banks with traditional business models must consider the possibilities of technology-driven digitalized financial services and coordinate common technological standards and banking functions to realize appropriate performance.

*… condensed*

**2.3. Relationship between technological innovation and performance**

Technology-driven financial service development approaches produce innovative outcomes for new business models, applications, and process or products [29]. Consequently, commercial banks initiate and accelerate various types of research and development activities for patent acquisition. For example, Wang et al. [30] suggested that the levels of fintech innovation outcomes (reduction in bank operating costs, service efficiency improvement, strengthened risk control capabilities, and enhanced customer-oriented business models) depend on the bank’s use of technological innovation. Wang et al. [25] empirically validated that a positive impact of IT investments on intellectual capital can lead to competitive advantage, contingent on firm type, size, positioning, and location.

However, because of the nature of patents which take time to come into effect, commercial banks often need government support and selective investments for desirable outcomes. For example, investment-driven technological innovations require time to actualize and may not show immediate results at initial financing [31]. Investments in internal projects alone do not directly lead to investment performance, and government regulations must be considered for a fintech boom to become apparent [32]. Moreover, Haddad and Hornuf [33] noted that the availability of Internet server security, mobile subscription, and labor force also affect the development of fintech-driven markets. Based on various efforts to understand the relationship between the development of intellectual capital (such as patents), banking competitiveness, and performance, internal and external factors must be considered [25].

There remains a critical decision support question regarding the financial services that technology must be strategically applied to in an orderly fashion to appropriately fulfill target customer needs from commercial banks anticipating the digital transformation of traditional business models.

**Reviewer#4, Concern #3** As the QFD methodology has been mentioned many times in this paper, it may be better to place the introduction of QFD in the front of Chapter 3.

**Author action:** The newly revised Section 3 includes an overall explanation about QFD analysis. The revision can be found in Section 3 on pp. 9 - 10 Line 205 - 213.

- **3. Research methodology and data analysis**

*… condensed*

QFD is a systematic framework originally developed for enhancing overall product and service design qualities by setting design targets based on the user’s needs and requirements [34]. The QFD application has proven useful in engineering and management for its usefulness in resolving design improvement solutions from a what and how perspective [35]. Specifically, for a complex problem related to technology development trends and changing dynamics in service characteristics, QFD can simplify decision-making problems. QFD has been applied to identify emerging robot technologies [35], innovative services in the healthcare industry [34], and technology implementation orders [37], thereby proving its usefulness and applicability in forecasting technologies.

*… condensed*

**Reviewer#4, Concern #4** This paper takes the four of the largest banks as examples, they are all excellent companies. Thus, how to convince readers the proposed framework can be applied to other financial service firms.

**Author action:** Traditional investment strategy of financial service firms are limited to the internal capabilities and knowledge of fintech applications. There is a strong need for generalizable approach to bridging the gap between the technological development trend and the applicability of specific domain (investment banking, commercial bank, etc). The proposed framework itself is generic, so it can be applied generally by adjusting WHAT and weight to the corresponding company. More specifically, if WHAT is adjusted according to the classification of the core service of the company and weight is assigned according to the importance of the service, a strategy specific to the company can be obtained. Therefore, the proposed approach enables to practitioners to make strategic decisions that aims to improve the productivity of fintech applications for desirable financial services. The application is demonstrated using four commercial banks in Korea but are not limited to other firms that are interested in taking evidence-based approach to identifying (i) key financial services, and (ii) financial technologies.

The revision can be found in Section 5 on p. 23 Line 418 – 428.

- **5. Conclusion**

*… condensed*

Three practical contributions are highlighted: First, it paves the way for advancing the knowledge of research gaps and practical solutions to incorporate financial technologies in the evaluation process for investment based on its management and operational goals. Banks can decide on investment strategies. In addition, the alignment between financial service development and fintech trends can be improved through the decision support framework proposed in this study. Most importantly, this framework can be generalized and applied to other firms. The outcomes of this research approach can support and enable practitioners to make strategic decisions to enhance the productivity of fintech applications in meeting financial services. To the best of our knowledge, this is the first study to use a QFD structure to identify fintech investment orders using objective technological trends based on patent information and subjective financial service trends based on expert information.

*… condensed*

**Reviewer#4, Concern #5** If possible, the overall length of the article can be reduced without affecting the expression of the author's core ideas.

**Author action:** We have thoroughly revised the manuscript and streamlined each section line-by-line. For example, Abstract and Introduction have been extensively revised to concentrate on the core idea of the manuscript.

**Reviewer#5 Concern #1:** The topic is too complex for global readers understanding. It needs to be updated. I suggest the topic to read as CTM and QFD analysis: Framework for Fintech Adoption Priority in Commercial banks.

**Author action:** We thank the reviewer for the recommendation. We decided to accept your suggestion, and we modified the title.

(Before)	Patent-based contextual topic modeling and QFD analyses for commercial banks’ adoption priority for fintech

(After)	CTM and QFD analysis: Framework for Fintech Adoption Priority in Commercial banks

**Reviewer#5 Concern #2:** Abstract: Bring out the purpose/objective of the study in the abstract. It’s missing. The abstract need to be reorganized bringing out the purpose of the study, methodology, finding and the value of the study. There seems to be a mixture up of issues. Give a summary of the study in the abstract to bring out the entire picture of the study in a summarized form.

**Author action:** As recommended, the abstract has been revised in the order of research objectives, methodologies, and results.

- As financial technology (fintech) is developing rapidly, many commercial banks experience difficulty deciding what kind of fintech to primarily focus on when managing their business. Owing to limited resources and assets, there is a practical need for guidelines for banks’ investments in fintech. This study provides a systemic procedure to identify promising fintech groups and their investment priorities. We propose a QFD-based decision support framework for banks by considering both aspects of the emerging fintech push identified using patent topic modeling and the market pull of banking services obtained from a survey of the literature and experts. An empirical application of the proposed QFD framework to major South Korean banks shows that transaction support technology, secure transactions, and trading platforms are the three most important fintech categories. The QFD results are utilized to guide individual banks for further investment strategies such as mergers and acquisitions, strategic partnerships, and spin-off operations. The proposed framework can be generalized and applied to other financial service firms.

**Reviewer#5 Concern #3:** In the introduction:

(i) Show a global perspective of the challenges facing banks in the choice and adoption of fintech. This will enrich the background bringing out the problem in a global context before you narrow down to Korean banks.
(ii) The proposed use of QFD framework in the introduction is inappropriate, instead it should be brought up in the methodology section in a systematic manner.
(iii) Create a section to discuss commercial banks in Korea in terms of their choice of fintech and adoption of the technology. This will help the readers and I, the global context understand the status of Korean banks in terms of fintech adoption.

**Author action:** We thank the reviewer for a constructive feedback and suggestions for improvements.

(i) As recommended, the global perspective of current challenges are highlighted through the environmental and economic concerns. The revision can be found in Section 1 on p. 3 Line 57 – 61.

- **1. Introduction**

*… condensed*

Major global banks, such as the Bank of America, JP Morgan, and Wells Fargo, hold many patented technologies. Nevertheless, these banks have not created innovative new business models using these technologies. This indicates that integrating new technologies and services is more complex than simply developing technology.

(ii) The section describing QFD framework is moved to Section 3 for better readability. The revision can be found in Section 3 on pp. 9 - 10 Line 197 – 213.

- **3. Research methodology and data analysis**

Based on a thorough literature survey, we propose a patent-based QFD framework by first identifying the areas for financial services with sublevels. Second, emerging technologies in the banking industry are classified into several areas by applying CTM to the abstracts of fintech-related patents. QFD is applied to identify the priorities of emerging fintech areas concerning the prioritized needs of the financial service categories. Empirical results were obtained by applying the proposed framework to patent data filed at the USPTO, along with a survey of major South Korean commercial banks. Fintech employment strategies are proposed based on this analysis.

QFD is a systematic framework originally developed for enhancing overall product and service design qualities by setting design targets based on the user’s needs and requirements [34]. The QFD application has proven useful in engineering and management for its usefulness in resolving design improvement solutions from a what and how perspective [35]. Specifically, for a complex problem related to technology development trends and changing dynamics in service characteristics, QFD can simplify decision-making problems. QFD has been applied to identify emerging robot technologies [35], innovative services in the healthcare industry [34], and technology implementation orders [37], thereby proving its usefulness and applicability in forecasting technologies.

*… condensed*

(iii) The discussions of the results in relation to the context of commercial banks in Korea are newly added on Table 8 in Section 4.2 on pp. 23 – 24.

- **4.2 Investment strategy for commercial banks**

*… condensed*

Finally, for Group III it is necessary to adopt conservative approaches in both investment and development strategies. Strategic partnership is recommended for the technology topics under this group, which will require a high level of collaboration for internal and external technology development for a desirable investment outcome. Results of the grouping and its implications are summarized in Table 8. We further interpret the abovementioned findings in the context of commercial banks in South Korea.

**Table 8.** Examples of degree of investment based on the partnership

|  | Full integration | Spin-off operations | Strategic partnership |
| --- | --- | --- | --- |
| Classification  (Examples) | Group I  (Topics 1, 3) | Group II  (Topics 2, 4, 5, 6, 11, 12) | Group III  (Topics 7, 8, 9, 10) |
| Technology details | Transaction security, Transaction support technology | Mobile transactions, trading platform, uncontacted financial service, Financial product valuation and design, hardware configuration, data analysis | Information and data processing, Personalized financial services, Insurance-related technology, Software for online services |
| Fintech service priority level (*y*-axis) | Moderate to High | Low to Moderate | Low |
| Fintech development status (*x*-axis) | Moderate to High | Moderate to High | Low |
| Development strategy characteristics | Time-sensitive involvements are required in both technology development and service integration as an immediate integration approach | The readiness of technology is considered relatively low. Instead of immediate application, the feasibility and assessments are recommended under collaborative efforts | Collaboration for both internal and external technology developments are recommended to for a synergistic performance |
| Degree of collaboration | High | Moderate | Low |
| Degree of investments | High | Moderate | Low |
| Examples in the South Korean context | Leading commercial bank in South Korea established Global Loyalty Network (“GLN”) which is a globally integrated platform that offers cross-border use of digital assets | Leading commercial bank in South Korea established a joint venture (JV) with medium size Tech company to lead the small and medium-sized business (SME) market by connecting financial ICT and technological competitiveness | Most large commercial banks in South Korea are incubating several technology startups for future growth |

**Reviewer#5 Concern #4:** In Literature review:

(i) Section 2.1 discusses mostly the challenges facing commercial banks. This section could fit well in the introduction section to aid in bringing up the problem under investigation. This section also concentrates more on discussing the benefits of fintech hence excluding the choices and strategies for adoption of fintech which are two major components of this study.
(ii) Theoretical literature missing. Introduce a section on theoretical review. Introduce a theory that guides this study.
(iii) Introduce a section on empirical literature and review studies relating to choose of fintech adoption in commercial banks, strategies of on the choice of fintech adopted, effect of patent based technologies and their effect. This will help in identifying research gaps.

**Author action:** The literature review has been restructured and modified to help the readers understand research background to this study for the practical needs (“a critical decision support question regarding for which financial services which technology must be strategically applied in an orderly fashion to appropriately fulfill target customer needs from commercial banks that are anticipating the digital transformation of traditional business models”) rather than theoretical basis. The revision can be found in Section 2 on pp. 5 – 9.

- **2. Literature review**

This section reviews research on fintech adoption and the relationship between innovation and performance from the perspective of commercial banks.

**2.1. Choices of fintech adoption**

Fintech and its adoption in the banking industry have brought innovations in various financial services, such as credit services, deposit services, financial market trading and brokerage, financial product advisory and retail sales, and transfer and global remittance [14–18]. Based on the predicated positive influence of fintech adoption in the commercial banking business model, various suggestions have been made regarding the scope of choices. For example, Thakor [14] proposed the following four services for fintech innovation: (i) credit, deposit, and capital-raising services (i.e., crowdfunding, lending marketplaces, mobile banks, and credit scoring); (ii) payments, clearing, and settlement services (i.e., mobile wallets and digital exchange); (iii) investment management services (i.e., e-trading and robo-advice); and (iv) insurance services (i.e., data-driven risk pricing and contracts).

.*… condensed*

**2.2. Strategies of the fintech adoption**

The general adoption or investment choice for which financial services should be improved depends on technology readiness and service strategies. Therefore, commercial banks must consider appropriate investment strategies for a desirable outcome given the intended scope of the services. Recent trends in commercial banks’ efforts to acquire and develop intellectual capital can be explained from two perspectives: internal efforts (i.e., hiring data scientists and operating internal projects for innovation) and external efforts (i.e., funding or participating in joint ventures or mergers and acquisition of technology companies). Brandl and Hornuf [23] differentiated investments from three perspectives: full integration of another company, strategic partnership between firms, and spin-off operations led by banks with traditional business models. They identified that banks with traditional business models must consider the possibilities of technology-driven digitalized financial services and coordinate common technological standards and banking functions to realize appropriate performance.

*… condensed*

**2.3. Relationship between technological innovation and performance**

Technology-driven financial service development approaches produce innovative outcomes for new business models, applications, and process or products [29]. Consequently, commercial banks initiate and accelerate various types of research and development activities for patent acquisition. For example, Wang et al. [30] suggested that the levels of fintech innovation outcomes (reduction in bank operating costs, service efficiency improvement, strengthened risk control capabilities, and enhanced customer-oriented business models) depend on the bank’s use of technological innovation. Wang et al. [25] empirically validated that a positive impact of IT investments on intellectual capital can lead to competitive advantage, contingent on firm type, size, positioning, and location.

However, because of the nature of patents which take time to come into effect, commercial banks often need government support and selective investments for desirable outcomes. For example, investment-driven technological innovations require time to actualize and may not show immediate results at initial financing [31]. Investments in internal projects alone do not directly lead to investment performance, and government regulations must be considered for a fintech boom to become apparent [32]. Moreover, Haddad and Hornuf [33] noted that the availability of Internet server security, mobile subscription, and labor force also affect the development of fintech-driven markets. Based on various efforts to understand the relationship between the development of intellectual capital (such as patents), banking competitiveness, and performance, internal and external factors must be considered [25].

There remains a critical decision support question regarding the financial services that technology must be strategically applied to in an orderly fashion to appropriately fulfill target customer needs from commercial banks anticipating the digital transformation of traditional business models.

**Reviewer#5 Concern #5:** In the methodology:

(i) Show the target population, how many commercial banks in Korea and justify the choice of the sample selected. Bring out the criteria used for selecting four firms for this study. Are they not operating under the same environment, have they not adopted the fin tech or what? I suggest that such a study is relevant if all firms are involved in the study sample unless there is a major reason for them to be excluded.
(ii) The study uses patents filed at USPTO up to the year 2020. There might be some more patents that could affect the choice of fintech adoption to date. Therefore, ensure that the study is updated to capture patents filed up to date to make the study more relevant with current trends.
(iii) To determine the weight of subjectiveness, brig out the criteria that was used to select the five practitioners/experts in each firm. Why choose five experts, is there any reason for this number?
(iv) In some section you claim to use 10 patents, in other section you use more patents in computation of weight of importance. There seems to be a disconnect between the two hence a clear justification id needed.
(v) In the methodology section you seem to have brought up the results. Concentrate on bringing out methodology used in section 3. The result fits well in section 4 where they are presented together with their discussion and interpretation.

**Author action:** We thank the reviewer for helpful feedback. We have incorporated explanations to the abovementioned concerns. We believe the newly revised version resolves concerns regarding (i) selection of case firms, (ii) selection of patent timeline, (iii) selection of subjective weight, (iv) clarification of patents number, and (v) conciseness of methodology.

(i) Four case firms are selected as they are the only nationwide company which offers overseas branches with global recognition. We also specifically included financial groups with headquarter located in Korea as to secure accuracy of practitioners’ weight evaluations which are carried out in Section 3.1. The explanation is added in Section 3 on p. 10 Line 214 – 223.

- **3. Research methodology and data analysis**

*… condensed*

As case companies for commercial banks, four of the largest banks ranked by asset value in South Korea were utilized in this study. Namely, Woori, Shinhan, KB, and Hana financial groups are the only nationwide companies headquartered in South Korea. These firms have approximately over 305 billion USD (400 trillion KRW) total assets with over 25,000 employees. They represent traditional and commercial banks in South Korea for their historic establishment, going back to the early 1900s, with hundreds of branches in South Korea and global branches in other countries. These firms offer retail, corporate, and international banking; credit card operations; foreign exchange; and other services. Other commercial banks with different headquarters but available in South Korea are excluded from the study for the accuracy of the classification process by field practitioners (Section 3.1).

(ii) For patents to become applicable and take effects in the practice, Broekel (2019) and Daiha et al. (2015) noted that the most recent years of data may be unreliable due to the time lag effect. For that reason, while the full coverage of recent patents would be beneficial for comprehensiveness, we believe that the selected time frame in current study fulfills the primary goal of representing technology development trend. To support the further development of current approach with the suggestion, we have recommended the comparison of results between comprehensive timeline versus recent timeline in the limited section. The suggestion can be found on Section 5 on p. 27 Line 478 – 480.

- *Broekel T. Using structural diversity to measure the complexity of technologies. Youn H, editor. PLoS ONE. 2019;14: e0216856. doi:*[*10.1371/journal.pone.0216856*](https://doi.org/10.1371/journal.pone.0216856)
- *Daiha K de G, Angeli R, de Oliveira SD, Almeida RV. Are Lipases Still Important Biocatalysts? A Study of Scientific Publications and Patents for Technological Forecasting. van Berkel WJH, editor. PLoS ONE. 2015;10: e0131624. doi:*[*10.1371/journal.pone.0131624*](https://doi.org/10.1371/journal.pone.0131624)
- *… condensed*

Third, the time that takes until commercialization from patent application should be considered when making strategic planning for the banks. For example, Broekel [44] and Daiha [45] noted a time lag for patent applications to come into effect.

(iii) To fulfill the primary goal of the study (providing a decision-making support framework for banks considering dual perspectives of fintech push and market pull of banking services), the weights are incorporated by the ‘importance, urgency, and subjective.’ In addition to objective weights (in this case, ‘importance’ and ‘urgency’), Pang et al. (2020) emphasized the integration need for the subjective weights such as experts’ preferences and determination. While the current stage of the study examines the results based on these weights, the follow-up study examines the anticipated impact and its sensitivity relating to the weight information at different levels. To remain within the scope of the study, we therefore do not test out diversified approaches to the sub-measure of the weights.

- *Pang J, Guan X, Liang J, Wang B, Song P. Multi-attribute group decision-making method based on multi-granulation weights and three-way decisions. International Journal of Approximate Reasoning. 2020;117: 122–147. doi:*[*10.1016/j.ijar.2019.11.008*](https://doi.org/10.1016/j.ijar.2019.11.008)

(iv) For the classification of fintech topics, over ten thousand of patents from period 2011 – 2020 are utilized. For specific topic labels, top ten patents with highest relation (ranging from 75% to 97%) were selected for the careful readings of abstracts.
(v) We thank the reviewer for a suggestion. To assist the readers in understanding the methodology and its application process, we have intended the methodology to be accompanied with analytic process. However, we do agree with you that the result should be more highlighted in Section 4 rather than Section 3. For that reason, we newly added Table 8 to condense the result and its implications from academic and managerial perspectives. Table 8 is in Section 4 on pp. 23 - 24.

**Reviewer#5 Concern #6:** Discussion: Discussion was ok, however there is no link to the reviewed literature. We should be able to see whether, the outcome of the study support or contradict prior empirical evidence.

(iv) **Author action:** We thank the reviewer for valuable feedback. The results, implications for academic and practical needs are further elaborated through Table 8 in Section 4 on pp. 23 - 24.

- **4.2 Investment strategy for commercial banks**

*… condensed*

Finally, for Group III it is necessary to adopt conservative approaches in both investment and development strategies. Strategic partnership is recommended for the technology topics under this group, which will require a high level of collaboration for internal and external technology development for a desirable investment outcome. Results of the grouping and its implications are summarized in Table 8. We further interpret the abovementioned findings in the context of commercial banks in South Korea.

**Table 8.** Examples of degree of investment based on the partnership

|  | Full integration | Spin-off operations | Strategic partnership |
| --- | --- | --- | --- |
| Classification  (Examples) | Group I  (Topics 1, 3) | Group II  (Topics 2, 4, 5, 6, 11, 12) | Group III  (Topics 7, 8, 9, 10) |
| Technology details | Transaction security, Transaction support technology | Mobile transactions, trading platform, uncontacted financial service, Financial product valuation and design, hardware configuration, data analysis | Information and data processing, Personalized financial services, Insurance-related technology, Software for online services |
| Fintech service priority level (*y*-axis) | Moderate to High | Low to Moderate | Low |
| Fintech development status (*x*-axis) | Moderate to High | Moderate to High | Low |
| Development strategy characteristics | Time-sensitive involvements are required in both technology development and service integration as an immediate integration approach | The readiness of technology is considered relatively low. Instead of immediate application, the feasibility and assessments are recommended under collaborative efforts | Collaboration for both internal and external technology developments are recommended to for a synergistic performance |
| Degree of collaboration | High | Moderate | Low |
| Degree of investments | High | Moderate | Low |
| Examples in the South Korean context | Leading commercial bank in South Korea established Global Loyalty Network (“GLN”) which is a globally integrated platform that offers cross-border use of digital assets | Leading commercial bank in South Korea established a joint venture (JV) with medium size Tech company to lead the small and medium-sized business (SME) market by connecting financial ICT and technological competitiveness | Most large commercial banks in South Korea are incubating several technology startups for future growth |

**Reviewer#5 Concern #7:** Conclusion should be done before implications to enhance the flow of ideas.

**Author action:** As recommended, the order of sections has been revised. The changes can be found in Section 5 on pp. 24– 27.

**Reviewer#5 Concern #8:** Check the entire document for typo and grammatical errors.

**Author action:** We thank the reviewer for a careful attention to the details. The manuscript has been thoroughly checked for grammar errors by professionals from the Cactus Communication.
