# Supplementary material for: CTM and QFD analysis: Framework for fintech adoption priority in commercial banks
Source: PLoS One. 2023 Nov 1;18(11):e0287826. doi: 10.1371/journal.pone.0287826 (PMC10619835; doi:10.1371/journal.pone.0287826)
Supplement: Attachment — Submitted filename: Response to Reviewer_20230612.docx [file pone.0287826.s003.docx]

**Original Manuscript ID:** PONE-D-22-32567

**Original Article Title:** “Patent-based contextual topic modeling and QFD analyses for commercial banks’ adoption priority for fintech”

**Revised Article Title:** “CTM and QFD analysis: Framework for Fintech Adoption Priority in Commercial banks”

**To:** PLOS One Access Editor

**Re:** Response to reviewers

Dear Editor,

Thank you for allowing a resubmission of our manuscript, with an opportunity to address the reviewers’ comments.

We are uploading (a) our point-by-point response to the comments (below) (*Response to reviewers*), (b) an updated manuscript with blue colors indicating changes (*Revised Manuscript with Track Changes*), and (c) a clean updated manuscript without blue color (*Manuscript).*

We hope that this revision fully reflected the major concerns of the reviewers.

Best regards,

D. Kang and S. Sohn

**Reviewer#5 Concern #1:** In the introduction:

A global perspective of the challenges facing banks in the choice and adoption of fintech has been incorporated as suggested previously hence the background has been enriched bringing out the problem in a global context. Challenges facing Korean banks has not been discussed in the background in terms of fintech adoption. This is very vital for this study. I suggest the Author to create a section in the background to discuss commercial banks in Korea in terms of their choice of fin-tech and adoption of the technology. This part has not been done as was suggested previously. hence with the current version in the introduction, the reader is not able to conceptualize the situation of fintech adoption in the context of Korean Banks

**Author action:** We thank the reviewer for a constructive feedback and suggestions for improvements.

As recommended, Challenges facing Korean banks are highlighted. The revision can be found in Section 1 on p. 3 Line 62 – 74.

- **1. Introduction**

*… condensed*

The challenges facing Korean banks are more threatening than those of global banks. Mobile applications created by Fintech-based startups with large-scale investments have secured monthly active users. They exceed those of commercial bank mobile apps in a short period of time, and the gap is gradually widening [7]. A greater threat is the emergence of Internet-based banks. In particular, the rapid asset growth of Kakao Bank, a subsidiary of Kakao, which controls more than 95% of the Korean mobile messenger market, could be a factor in declining market share of banking industry. Korean commercial banks that lack IT-based technology and manpower are in a rush to imitate the rapid service launches with modern technologies of fintech-based startups and internet-only banks [8]. Therefore, Korean commercial banks are aware that it is essential to secure fintech to become a leader, not a follower. However, as can be seen from the examples of major global banks that have sufficient technology, more complex considerations are needed because simply securing technology does not create nor guarantee a new business model.

**Reviewer#5 Concern #2:** In Literature review: The empirical literature was well updated to capture the choice of fintech in banks and the strategies adopted. However, the Author has not indicated the theories that this study is anchored to. this is very vital. This was previously suggested. Let the Author briefly relate this study to an existing theory by including a section on theoretical literature.

**Author action:** As recommended, the newly revised literature section discusses the technology adoption theories which provide a foundation to our research on fintech adoption. The revision can be found in Section 2 on p. 9 Line 186 – 196.

- 2.3. Relationship between technological innovation and performance

Theoretical foundations of technology adoption research share two viewpoints: adopters (user-level) and service provider (firm-level). The most prominent theories include theory acceptance model (TAM), unified theory of acceptance and user of technology (UTAUT), diffusion of innovation (DOI), and dynamic capability to name a few [30–33]. Among the theories, firm-level viewpoint often relies on DOI and dynamic capability [33,34]. Similarly, financial service firms (including both traditional and internet-only banks) anticipate the growth of capabilities leading to the innovation outcome and the potential performance improvement [35]. The dynamic capability view elaborates how firms can utilize capabilities and the external trend to gain competitive advantage in the market [36]. Based on the view, the technology adoption in financial services can be regarded as a modification or a complete renewal of existing service capabilities to fulfill the market’s needs.

**Reviewer#5 Concern #3:** In the methodology:

(i) As previously suggested, the author ought to have indicated the number of commercial banks operating in south Korea, and further justify the choice of the four selected. as it is in this revised version, the reader is not able to understand the choice of the four banks well. as a matter of fact, all banks operating in a given country are regulated by the central banks in that country as far as regulations and operations is concerned. it would have been appropriate to show the target population and how the sample was arrived at.
(ii) As previously suggested, the Author should separate methodology and results. Bring out methodology clearly. let the results be in a different section. may be you can rename section 4 as Results and Discussion.

**Author action:**

(i) As recommended, I add reasons for selecting four banks for study. The revision can be found in Section 3 on p. 11 Line 243 – 248.

- **3. Research methodology and data analysis**

*… condensed*

There are 19 commercial banks in Korea. Among them, 5 are special purpose banks owned by the government, 6 are local banks that can operate only in the provinces, 3 are Internet based banks, and 2 are foreign banks headquartered overseas. The four banks selected for the study are privately owned, nationally operational banks. In other words, only the four banks selected in the study are commercial banks that operate freely without any particular purpose.

(ii) As suggested, QFD results have been moved to Section 4, and Section 4 has been renamed to ‘Results and discussion’.

**Reviewer#5 Concern #4:** In the Discussion:
As previously suggested, let the discussion be linked to the literature. This has not been done.

**Author action:** We thank the reviewer for valuable feedback. The newly revised conclusion section discusses the findings and contributions in relation to the literature. The revision can be found in Section 5 on p. 25 Line 445 – 472 and Line .486 - 487

- **5. Conclusion**

*… condensed*

This study primarily aimed to resolve the absence of a decision framework for investment in technology, specifically from the perspective of commercial banks. Rooted in the technology adoption theory, customer-centric financial services were classified based on the integrative views of the literature and field practitioners. Then, the emerging topics of technological trends were identified using a patent database. CTM and QFD analyses were applied to extract technology investment priorities and recommendations for acquisition strategies for each fintech topic.

This study has significant implications according to the findings. First, technology investment priorities must be determined based on the overall financial service strategy. R&D and technology investments are likely to lead to superior innovation capabilities which can thereby enhance new core competency based on the theory of dynamic capabilities [36,44]. However, the recent trends in prioritizing technology investment have mainly investigated the investment productivity utilizing efficiency measure approaches such as data envelopment analysis [53]. Our findings instead highlight the importance of the identification of service strategy prior to deciding the investment priorities as the results are differentiated based on the urgency of service development. Banks must decide on appropriate investment strategies and develop internal and external resources instead of depending on the technology advancement for the investment decision.

Second, our findings reshape technology acquisition strategies of commercial banks. Insights obtained from our study includes (1) transaction support technology, secure transactions, and trading platforms are commonly evaluated as the most critical technology topics; and (2) commercial banks are recommended to make investment strategies such as M&As, strategic partnerships, and spin-off operations considering the number of patents, importance of technology, and size of patent-owned fintech firms. Despite various literatures emphasizing the importance of technology-empowered personalized services [54], our study well aligns with Cao et al.’s [21] study in that transaction security is the utmost priority. This may be due to the high level of collaboration required for security technology. Thus, the proposed approaches is novel in comparison to the recent trend in emphasis of the fintech adoption (without consideration of the investment orders), our approach demonstrates the practical ways to reflect both.

*… condensed*

This appears to be well aligned with the global trend in fostering the ecosystem of fintech startups and commercial banks in the financial sector [55].
